# History of familial adult myoclonus epilepsy/benign adult familial myoclonic epilepsy around the world

**DOI:** 10.1111/epi.17519

**Published:** 2023-02-10

**Authors:** Samuel F. Berkovic, Pasquale Striano, Shoji Tsuji

**Affiliations:** ^1^ Department of Medicine, Epilepsy Research Centre University of Melbourne Heidelberg Victoria Australia; ^2^ Pediatric Neurology and Muscular Diseases Unit Giannina Gaslini Institute, Scientific Institute for Research and Health Care Genoa Italy; ^3^ Department of Neurosciences, Rehabilitation, Ophthalmology, Genetics, Maternal and Child Health University of Genoa Genoa Italy; ^4^ Department of Neurology University of Tokyo Hospital Tokyo Japan; ^5^ Institute of Medical Genomics International University of Health and Welfare Chiba Japan

**Keywords:** familial epilepsy, genetics, myoclonus epilepsy

## Abstract

Familial adult myoclonus epilepsy/benign adult familial myoclonic epilepsy (FAME/BAFME) has emerged as a specific and recognizable epilepsy syndrome with autosomal dominant inheritance found around the world. Here, we trace the history of this syndrome. Initially, it was likely conflated with other familial myoclonus epilepsies, especially the progressive myoclonus epilepsies. As the progressive myoclonus epilepsies became better understood clinically and genetically, this group began to stand out and was first recognized as such in Japan. Subsequently, families were recognized around the world and there was debate as to whether they represented one or multiple disorders. Clarification came with the identification of pentanucleotide repeats in Japanese families, and FAME/BAFME was quickly shown to be due to pentanucleotide expansions in at least six genes. These have geographic predilections and appear to have been caused by historically ancient initial mutations. Within and between families, there is some variation in the phenotype, explained in large part by expansion size, but whether there are features specific to individual genes remains uncertain.

**Key Points**

- FAME/BAFME is now recognized around the world as an autosomal dominant epilepsy syndrome with a specific phenotype
- Probable examples of FAME/BAFME were published >50 years ago, but clear recognition began in Japan in the 1990s
- Initially, the disorder was conflated with other familial myoclonus epilepsies, especially progressive myoclonus epilepsies
- Although the phenotype is characteristic, some variation is described between and within families
- The 2018 discovery of expanded pentanucleotide repeats in various genes will facilitate diagnosis and phenotype–genotype correlations

This syndrome, with its characteristic autosomal dominant inheritance, adolescent/young adult onset of “cortical tremor,” and myoclonus and tonic–clonic seizures, was first conceptualized by Japanese authors in the early 1990s. It was not appreciated outside Japan until some years later. The complex literature is confounded by multiple names; no less than 10 terms were identified in the 2019 review of van den Ende et al.^1^ The most commonly used names are benign adult familial myoclonic epilepsy (BAFME), favored by Japanese authors^2^; familial adult myoclonus epilepsy (FAME) generally utilized by epileptologists^3^ and in Online Mendelian Inheritance in Man; and familial cortical myoclonic tremor with epilepsy, favored by movement disorder specialists.^4^ In this historical review, we try to utilize the terms as published in the key papers; we use “FAME/BAFME” when referring generically to the disorder.

Here, we trace the history of “FAME/BAFME” over two eras. The first era (1891–1990) involved clarification of the nosology, phenotypes, and genetics of the familial myoclonus epilepsies, the majority of which we now recognize as the genetically heterogeneous group of progressive myoclonus epilepsies (PMEs). Retrospectively, it is likely that FAME/BAFME families were contained in some of those reports.

The second era (1990–present) involves the definitive descriptions of BAFME, initially from Japan, and the subsequent clinical recognition outside Japan. Debate ensued as to whether there were a number of different clinical entities. This was further complicated by reports of different linkage loci in various cohorts, and descriptions of genetic variants in certain families, which later were determined to be benign polymorphisms. Clarification began with the landmark 2018 report of Ishiura et al.^5^ describing pentanucleotide expansions in Japanese families, which opened the door to a series of molecular studies around the world.

## 1 FIRST ERA (1891–1990)

Nikolaus Friedreich (1881) is often credited with the first clear description of myoclonus, reporting “paramyoclonus multiplex” in a single patient who had multifocal myoclonus^6^ and is generally now thought to have suffered from the nonepileptic disorder, myoclonus dystonia. Myoclonus and epilepsy were, however, reported in 19th century writings from England and France as reviewed by Genton et al.^7^

*‐Familial* myoclonus epilepsy was first clearly described by Heinrich Unverricht^8^, ^9^ from Magdeburg, in patients from Estonia. Subsequently, Hermann Lundborg in 1903 in Sweden performed a genealogical study of “Unverricht's Myoklonie” and established its autosomal recessive mode of inheritance.^10^ This is now known as Unverricht–Lundborg disease, the prototypic myoclonus epilepsy.

Early autopsy studies suggested that there were three broad pathological groups among these familial myoclonus epilepsies: (1) Lafora type; (2) lipid storage type; and (3) “degenerative” type, with neuronal loss and gliosis.^11^, ^12^ Attempts were made at clinicopathologic and genetic correlations, but the literature was very confusing and inconsistent. Diagnosis during life was rarely possible. From the 1980s, with more thorough clinical studies and advances in minimally invasive pathological diagnosis (e.g., skin biopsy), biochemistry, and subsequently molecular genetics, diagnosis in life of the major disorders of Unverricht–Lundborg disease, Lafora disease, neuronal ceroid lipofuscinoses, mitochondrial diseases, and other conditions became possible.^13^, ^14^

**FIGURE 1:**
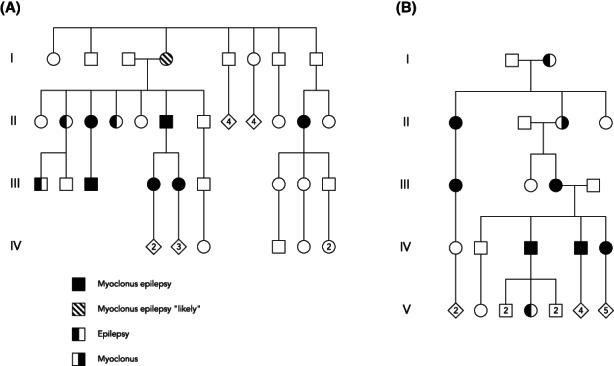
Two redrawn historical pedigrees probably representing early descriptions of familial adult myoclonus epilepsy/benign adult familial myoclonic epilepsy. (A) An autosomal dominant pedigree initially reported in 1965 by Vogel and Diebold.^18^, ^19^, ^20^ (B) French family described in detail in 1967 by Castaigne et al.^22^

There was also a group of families described in the early literature with phenotypes that were milder than classical PMEs. Onset was often in late teenage or adulthood, seizures were infrequent or absent, inheritance was autosomal dominant, and progression was slow or absent. Some were described as “familial/hereditary essential myoclonus.”^15^, ^16^ In retrospect, this group was likely etiologically heterogeneous, with some families having the nonepileptic disorder of myoclonus dystonia, often due to pathogenic variants in sarcoglycan epsilon^17^ and some probably having FAME/BAFME. Evidence for the latter conclusion comes from reviewing the extensive clinicopathologic–genetic analysis of Vogel and Diebold, who classified 57 cases of PME into four forms,^18^, ^19^, ^20^ of which one was a rare dominant form of myoclonus epilepsy that was slowly progressive, associated with “degenerative changes,” named by them as the “Hartung type”; the clinical descriptions and some pedigrees suggest FAME/BAFME to us (Figure 1), although the original report of Hartung^21^ described a more severe and probably different disorder. Similarly, in 1967, Castaigne et al. described a five‐generation pedigree with “familial progressive myoclonus epilepsy” from the Salpêtrière in Paris^22^ that was also very likely FAME/BAFME on review of the data (Figure 1).

In the Japanese literature, Wakeno^23^ described a family with tremor and epileptic seizures in 1975, and seven such families with “heredofamilial tremor and epilepsy” were reported by Kudo et al. in 1984.^24^ These publications went essentially unrecognized outside of Japan.

## 2 SECOND ERA (1990–PRESENT)

In the early 1990s, a series of further Japanese papers elucidated BAFME in more detail,^2^, ^25^, ^26^ including an important 1990 paper in English by Ikeda et al. that provided a detailed neurophysiological analysis of cortical tremor; the key patient was from a BAFME family.^27^ This paper focused on neurophysiology, rather than the clinical syndrome and inheritance; in retrospect, this may explain why there was not more widespread clinical recognition of the disorder outside of Japan after this publication.^27^ A number of other Japanese reports followed in the latter 1990s (reviewed in Naito and Oyanagi^28^). Japanese authors discussed the differential diagnosis from “familial essential myoclonus”^25^ and from dentatorubral–pallidoluysian atrophy, another dominant disorder with myoclonus and seizures that is relatively prevalent in Japan.^28^, ^29^, ^30^, ^31^ In 1999, linkage of Japanese BAFME families to chromosome 8q was reported.^32^, ^33^

Publication of non‐Japanese families began in 1998 with Elia et al.,^34^ who described an Italian family with dominant cortical tremor and associated intellectual impairment in some members. A five‐generation Turkish family with migraine in association with FAME/BAFME was reported in 2000.^35^ In 2001, Guerrini et al. described a Tuscan family with features they recognized as similar to FAME/BAFME. However, they regarded it as a different disorder because of additional focal seizures and mild intellectual disability in some subjects, as well as linkage to chromosome 2, differing from the chromosome 8q linkage in Japanese BAFME families.^36^ Similarly, absence of linkage of a French family^37^ and of a Dutch family^38^ to chromosome 8q was reported, highlighting the genetic heterogeneity of FAME/BAFME. Two further Italian families were published with evidence supporting linkage to chromosome 2.^39^, ^40^

In 2005, van Rootselaar et al. summarized the features of 41 Japanese and seven non‐Japanese families, highlighting that tremor was the initial symptom, with subsequent appearance of myoclonus and tonic–clonic seizures in some, with rare focal seizures. Age at onset varied from 10 to 60 years, with the mean in the 20s. In addition to intrafamily variation, there seemed to be some variation between families, and it was suggested that European cases were more severe than Japanese cases. Electrophysiological studies showed features of cortical reflex myoclonus. The course was generally benign; cognitive decline was an occasional feature. They concluded that the various reports from Japan and Europe represented one disease spectrum.^4^

The disorder became more widely recognized, and in 2018 van den Ende et al. reviewed studies containing 126 families worldwide. In addition to families from Japan, Western Europe, and Turkey, there were reports of pedigrees from China, India, Thailand, Australia/New Zealand, Spain, and South Africa.^1^ The previously recognized clinical features were confirmed. Mild cerebellar features were occasionally noted, and the presence of cognitive decline was reported in some families. A variety of other psychiatric and neurological features were reported in some families, but none with a consistency suggesting that they were truly part of the FAME/BAFME phenotype, rather than incidental comorbidities. Clinical anticipation was reported in some Japanese families,^41^, ^42^ but without very large pedigrees, and because of possible ascertainment bias, such observations were cautiously interpreted in the absence of a molecular mechanism.

Large autosomal dominant families are useful for linkage analysis and, in addition to the 8q locus in Japanese families (FAME1) and the pericentomeric chromosome 2 locus (FAME2) in Italian and other European families, additional loci were reported on 5p (FAME3)^43^ and 3q (FAME4).^44^ Haplotype analyses of families mapping to the FAME2 locus suggested allelic heterogeneity, with at least four distinct founders.^45^ Vigorous attempts were made to identify the underlying genes by searching for pathogenic variants using conventional sequencing methods. Missense and other variants were described in a number of potentially relevant genes (reviewed in van den Ende et al.^1^), but these now appear to be benign polymorphisms.

Ishiura et al.,^5^ in an extensive series of experiments using fine linkage mapping, whole genome sequencing, repeat‐primed polymerase chain reaction, Southern blot analysis, and single‐molecule real‐time sequencing of cloned mutant alleles in bacterial artificial chromosomes, demonstrated abnormal expansions of TTTCA and TTTTA repeats in intron 4 of *SAMD12* on chromosome 8q in 49 Japanese families with BAFME. Two families lacked the expansion in *SAMD12*, but one showed the same TTTCA and TTTTA expansions in *RAPGEF2* (Ch 4q) and the other in *TNRC6A* (Chr 16p). The age at onset inversely correlated with the expansion size, providing a molecular explanation for clinical anticipation.^5^

Subsequently, there was rapid growth in knowledge, with recognition of related pentanucleotide expansions in several genes, often with particular geographic distributions. The same pentanucleotide expansions were subsequently found in families of European origin mapping to chromosome 2 (*STARD7*)^46^ and chromosome 5q (*MARCH6*)^47^ and in *YEATS2* in Thai families.^48^ The FAME1 expansion in *SAMD12* was subsequently found in Chinese, Indian, Sri Lankan, and Thai families (Table 1).^49^, ^50^, ^51^, ^52^

**TABLE 1:** Worldwide distribution of FAME/BAFME types (adapted from Ishiura and Tsuji^54^).

OMIM designation	Locus	Gene	Geographic distribution	Number of known families (approximate)
FAME1	8q24.12	*SAMD12*	Japan, China, India, Sri Lanka, Thailand	>120
FAME2	2q11.2	*STARD7*	Italy, France, Spain, Australia/New Zealand, Iraq, Israel, Syria, South Africa	22
FAME3	5p15.2	*MARCH6*	France, Germany, The Netherlands	4
FAME4	3q27.1	*YEATS2*	Thailand	1
FAME6	16p12.1	*TNRC6A*	Japan	1
FAME7	4q32.1	*RAPGEF2*	Japan	2

Abbreviations: BAFME, benign adult familial myoclonic epilepsy; FAME, familial adult myoclonus epilepsy; OMIM, Online Mendelian Inheritance in Man.

The size of the expansions varies between family members and correlates with clinical anticipation. Families in diverse geographical areas share haplotypes, and it is estimated that the original mutations for *SAMD12* occurred approximately 17 000 years ago.^51^, ^53^ New mutations and sporadic cases have not been identified by molecular methods to date, but this is an area worthy of further study. Further studies are also required to clarify whether there are gene‐specific variations in phenotype.

The pathogenesis of FAME/BAFME remains to be clarified, but initial evidence favors a toxic gain‐of‐function mechanism.^54^ We now have the new mystery of how similar expansions in different genes, with apparently diverse functions, result in essentially the same disorder.

## AUTHOR CONTRIBUTIONS

Samuel F. Berkovic wrote the first draft. All authors critically reviewed the manuscript and approved the final version.

## CONFLICT OF INTEREST STATEMENT

None of the authors has any conflict of interest to declare.
